# How to grow a successful – and happy – research team

**DOI:** 10.1186/s12966-019-0907-1

**Published:** 2020-01-14

**Authors:** Kylie Ball, David Crawford

**Affiliations:** Institute for Physical Activity and Nutrition, Deakin University, Geelong, VIC Australia

**Keywords:** Academic wellbeing, Research culture, Leadership, Happiness

## Abstract

Changing academic landscapes, including the increasing focus on performance rankings and metrics, are impacting universities globally, contributing to high-pressure environments and anxious academic staff. However, evidence and experience shows that fostering a high performing academic team need not be incompatible with staff happiness and wellbeing.

## The changing academic landscape

Global academic rankings have become a key indicator of the success of universities. Ranking systems are used by universities to mark improvement over time and in comparison to other institutions, and as evidence of progress when requesting government funding. They are also used by consumers to evaluate higher education opportunities [9]. This intensified focus has led to pressure on universities to improve their performance and position in rankings tables [3].

Reputation and research citations account for the majority of the rankings. Advice on improving rankings has hence focused on strategies such as hiring research ‘stars’ and increasing research volume; that is, on strategies for growing research. Relatively little attention has focused on growing researchers. For example, a Times Higher Education list of 20 tips for improving rankings included “no pain no gain” (in making tenure decisions) as one tip, yet featured only two fleeting references to strategies focused on nurturing academics [4].

In addition to the increased pressures associated with achievement of research metrics and rankings, other expectations of academics have increased over the last decade. For example, the impact agenda demands research that makes a difference, that is engaged with industry, community or political partners. Such research requires new tasks, networks and skillsets for which many academics are not trained. At the same time, government funding for research and research staff has declined in many countries, including North America, the UK and Australia. The academic workforce has become increasingly characterised by short-term contracts, workforce casualisation and lack of longer-term job security and pathways. In some countries the sector has also been impacted by the rise of national assessments of research quality (e.g. through the UK Research Excellence Framework; the Excellence in Research for Australia Framework; or the Netherlands’ Standard Evaluation Protocol). The time required to both prepare and assess submissions for these exercises is substantial.

What are the impacts of the changing academic landscape on staff? Academics appear to be generally unhappy. A recent (June 2019) Google search showed the following suggestions based on common searches for ‘academia is’:

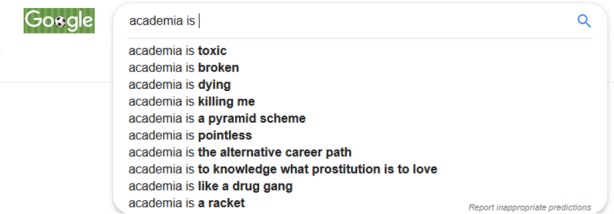



Universities have been described as “Anxiety Machines” [6]. Mental health problems are at levels described as ‘epidemic’, with one study showing staff referrals for counselling increasing by between 50% to over 300% between 2009 and 2016 [6]. These problems have been linked at least in part to excessive workloads and demands in an increasingly competitive culture.

## Are high performance and happiness incompatible?

Highly successful academic teams require intensive focus and effort; this may be perceived as incommensurate with a happy workplace. A web scan of publically available university strategic plans shows that many focus heavily on performance yet lack consideration of strategies that can foster enhanced staff happiness and wellbeing. Possibly, the latter are generally not considered as important in the ‘rankings race’.

Happiness makes people more productive at work. One study found that happiness resulted in a 12% increase in employee productivity, whereas unhappy workers were 10% percent less productive than average [7]. Psychological wellbeing is also positively correlated with staff retention [10], which is particularly important in a university context considering the long lead times required to build a successful research program. Promoting happiness among university staff is a worthy goal.

Our experience shows that fostering a high performing academic team is not incompatible with staff happiness. Our School has been twice ranked number one globally in its discipline (Academic Ranking of World Universities (ARWU) 2016, 2017; 3rd in 2018) (www.shanghairanking.com). In addition, our staff survey and Early-Mid Career Researcher (EMCR) evaluation results show that our staff feel happier and more supported when benchmarked against the broader university sector. Staff survey results show, for example, that on measures of feeling supported in research (‘I am given enough support to achieve my research goals’), our staff score 27 percentage points higher than the sector average. On indicators of wellness (‘I feel emotionally well at work’; ‘I am able to manage stress at work’), our staff score 5–8 percentage points higher, and on various measures of job satisfaction our staff score 4–6 percentage points higher than the sector average. Similarly, evaluation showed that our School’s EMCR initiatives were associated with increased career satisfaction and morale, and decreased perceived distress.

We’ve implemented initiatives that focus on people and their happinness, as well as performance. These include a dedicated role with oversight of the development, care and support of early- and mid-career researchers through a range of mentorship, face-to-face and virtual support channels.

Academic workloads are an often-cited source of stress, and evidence shows that researchers who are happy are those who have time (particularly unfragmented time) to do research [2]. We designed strategies to create uninterrupted time, including batched teaching within semesters, and an ‘internal sabbatical’ program to free up extended time for staff to advance their research without teaching commitments.

Other strategies we have implemented include providing flexibility to allow staff to work to their strengths; adhering to publication guidelines whereby authorship is truly representative of inputs; greater recognition of not just winning funding but having a go; and encouraging and modeling a team-based approach in which individuals are not just working in a group, but encouraging colleagues to look out for and after each other at all levels. This is consistent with evidence that shows building social interactions and high-quality connections fosters knowledge-intensive workers’ happiness at work [8]

Other leading groups have adopted similar initiatives. The University of Ghent has recently initiated an approach that directly addresses the signs of increasing pressure on academic staff. Their new model emphasises staff development; collegiality rather than competitiveness; reduced reliance on quantitative metrics; and being a ‘caring’ employer (https://www.u4network.eu/index.php/news/2707-ghent-university-is-changing-course-with-a-new-career-model-for-professorial-staff-press-release-ghent-17-september-2018).

A successful and happy research team does not just happen. To grow research, you need to grow and nurture researchers. This means more than the usual professional development opportunities, annual performance meetings, perks such as subsidised gyms or childcare and performance bonuses. In our experience, leadership is the key. Modern reseach leadership is not about being the most successful or highest-cited academic - it is about a commitment to supporting and developing others, and creating an environment in which they can succeed. Leadership styles which focus on people have greater impact on happiness at work compared to transactional leadership styles [8].

## Nurturing a successful and happy research team

Based on our experience and the existing evidence, we advocate a shift in academic leadership, cultures, training and practice, towards a kinder, more people-focused approach. Our top tips for academic leaders interested in growing successful and happy research teams include:

### Focus on individuals and their career development

Facilitate career conversations and pathways that offer opportunities for development. Set up one-to-one mentoring relationships and a culture in which senior staff are expected to mentor not only their direct reports but others from whom they stand to gain no ‘KPI’ benefit.

### Prioritise staff happiness and wellbeing

Seek ways to reduce, not increase, staff workloads. Allow staff to set their own schedules and enable uninterrupted times for scholarly contemplation and research. Encourage and model a balanced perspective, with sensible working hours, in which work is not the only or most important component of life. Support collegial social events.

### Foster a culture of kindness

Academia is very good at teaching us to be critical, but neglects to skill staff in practices embodying kindness, despite evidence that such practices improve organisational effectiveness [1]. Encourage workplace kindness through expressing appreciation and gratitude to staff. Tell staff what you value about them. Share and celebrate staff successes, and foster a caring attitude to ‘rejections’. Embody a leadership approach that is both empathic and holistic, considerate to the ups and downs of non-work, as well as working life (for example through allowing greater flexibility in terms of staff work hours and locations during challenging life circumstances). Model respectful treatment of colleagues and compassionate support. When hiring new staff, seek those who are caring as well as competent.

### Challenge cultures and workplace models

  - in which all academics must be all things. Instead endorse essentialism [5]: the disciplined pursuit of less. Recognise that performance can be judged collectively across teams in which people have strengths across different domains – some are outstanding writers; others, great orators/teachers; others masters at industry engagement; others science communicators or policy influencers. Allow people flexibility to specialise and channel time and effort into making the best possible contribution to the most meaningful and important activities as part of a successful team.

### Advocate for a kinder approach to metrics

Foster collaborative, team-based approaches focused on improving the research quality of the team, rather than on metrics per se. For example, a focus on strengthening team members’ research skills and shared learning can both improve team ethos and raise collective rankings. We advocate that new rankings should incorporate a focus on the happiness of staff, given the strong case that happy, engaged workplaces foster both staff wellbeing and organisational productivity.

## Data Availability

N/A.
